# Incoherence and the balance of evidential reasons

**DOI:** 10.1007/s44204-023-00103-0

**Published:** 2023-09-21

**Authors:** Sebastian Schmidt

**Affiliations:** 1 Department of Philosophy, University of Zurich, Zurich, Switzerland; 2 African Centre for Epistemology and Philosophy of Science, University of Johannesburg, Johannesburg, South Africa

**Keywords:** Reasons-first, Suspending judgment, Incoherence, Evidence

## Abstract

Eva Schmidt argues that facts about incoherent beliefs can be non-evidential epistemic reasons to suspend judgment. In this commentary, I argue that incoherence-based reasons to suspend are epistemically superfluous: if the subjects in Schmidt’s cases ought to suspend judgment, then they should do so merely on the basis of their *evidential* reasons. This suggests a more general strategy to reduce the apparent normativity of coherence to the normativity of evidence. I conclude with some remarks on the independent interest that reasons-first epistemology might have within an evidentialist framework.

## Introduction

Consider one of the three cases that Eva Schmidt (2023b) presents as counterexamples to evidentialism—that is, the claim that all epistemic reasons for doxastic attitudes towards *p* are provided by evidence concerning *p*:

*History vs. Philosophy*

When Basna studies history, she comes to believe, based on her professors’ arguments, that the historical facts are relative to the historian who interprets historical sources. Basna later switches her focus to philosophy and, based on her philosophy professors’ arguments, she forms the belief that no facts are relative to anyone. Both beliefs are supported by sufficient evidence, which was provided, respectively, by Basna’s history professors and by her philosophy professors. Her evidence comprises both the professors’ expert testimony and the arguments with which she engages. At some point, Basna realizes that the two beliefs are inconsistent. (Schmidt, 2023b, 12)

In this version of the case, it is crucial that Basna is not *aware* of the contradiction until she has acquired both the belief that *p* (that historical facts are relative) and the belief that not-*p* (that they are not relative). As Schmidt puts it, “due to compartmentalization, she simply hasn’t compared notes, so to speak, and so has not yet weighed the arguments against each other” (Schmidt, 2023b, 12; see her fn. 17 on alternative versions of the case that work without compartmentalization). Crucially, in the moment *after* noticing her contradiction, and *before* having reevaluated her professors’ arguments, Basna should suspend judgment. According to Schmidt, *the fact that Basna holds contradictory beliefs* provides Basna with a non-evidential reason to suspend judgment that outweighs her evidential reasons for (dis)belief: in this moment, Basna has

a reason to take a step back from the whole mess that is her beliefs […] to reassess her whole epistemic situation with respect to the issue; to do so, she has to reopen the questions that she previously settled by believing the relevant propositions; but that is just to say that she has to suspend on these propositions. (Schmidt, 2023b, 8)

Clearly, Basna’s reason to suspend here cannot just be provided by *first-order* evidence, which seems to favor belief and disbelief, but not suspension. Furthermore, as Schmidt argues in detail, the incoherence between Basna’s beliefs is not in itself *higher-order* evidence: it is neither evidence *of* evidence, nor does it indicate any malfunctioning of Basna’s cognitive capacities.^1^ So, it seems that evidentialists cannot acknowledge epistemic reasons that are provided by facts about the incoherence of one’s own attitudes, for these are neither first- nor higher-order evidence. Rather, they are facts about the relationship between one’s attitudes.

Schmidt presents a distinctive new challenge for evidentialism. Incoherence, just like evidence, pertains to the truth of what is believed. Incoherence-based reasons would therefore force epistemologists to go beyond evidence in a way that can acknowledge the distinctively epistemic or truth-related flavor of epistemic reasons. In this way, Schmidt’s challenge can seem more pressing than Mark Schroeder’s (2012b, 2021) arguments that the practical costs of error can be non-evidential epistemic reasons against belief: the practical flavor of Schroeder’s epistemic reasons will more easily raise the eyebrows of evidentialists. Dialectically, Schmidt’s cases are therefore *prima facie* more challenging than Schroeder’s arguments.

Yet, evidentialists should not give in too quickly to Schmidt’s challenge, for any plausible evidentialist account of epistemic reasons must acknowledge the different kinds of evidence—first-order and higher-order—that can affect the epistemic rationality of a belief (Schmidt, 2023b, 4–7). This provides the material for a convincing evidentialist reply. In particular, I will argue that incoherence has no role to play in justifying beliefs, nor in motivating a rational agent: incoherence-based reasons are normatively superfluous.

I focus on the case of *History vs. Philosophy* introduced above, because I think our intuition that Basna should suspend judgment is clearer than in the other two cases presented by Schmidt—which concern lottery propositions and epistemic akrasia. However, as soon as we see why this case does not refute evidentialism, we will also see a general strategy for evidentialists to argue that these other counterexamples fail as well.

I first argue that the incoherence-based reason in *History vs. Philosophy* is normatively superfluous: the balance of evidential reasons alone tells Basna to suspend judgment (Section 2). I then show how this can help evidentialists to deal with Schmidt’s two other cases (Section 3). What arises is a more general strategy to reduce the apparent normativity of incoherence to the normativity of evidence. I describe this strategy in the conclusion (Section 4) and suggest that reasons-first epistemology is still attractive for evidentialists.

## Incoherence-based reasons are normatively superfluous

It is indeed difficult to see, at first glance, what evidentialists should say about reasons for suspending judgment. Schroeder puts the intuitive problem for evidentialism as follows:

Why is it that reasons to withhold cannot be evidence? It is because the evidence is exhausted by evidence which supports p and evidence which supports ~p. But the evidence which supports p is reason to believe p, and the evidence which supports ~p is reason to believe ~p. Consequently the reasons to withhold must come from somewhere else. So they cannot be evidence. (Schroeder, 2012a 276–277)

This gives rise to a more systematic problem if we focus on cases of *tied evidence*: where your (total) evidence for *p* and for not-*p* is roughly equally strong. It seems that evidentialists must say that your epistemic *reasons* for believing *p* and for believing not-*p* are (roughly) equally weighty as well—since evidence for *p* just is what gives us reasons for believing *p* and evidence that not-*p* is what gives us reasons for disbelief. Evidentialists now must add that your reason to suspend judgment *is weightier than* either the reasons for belief or the reasons for disbelief. For otherwise, they could not make it intelligible why one ought to suspend judgment in cases of tied evidence. Yet, it is not clear how evidence could still provide such reasons to suspend.^2^

Schmidt herself offers the beginning of a reply to this problem. She acknowledges that, on a broad conception of evidence, higher-order evidence can provide reasons for suspension. More specifically, she mentions that “higher-order evidence that the subject’s evidence concerning *p* does not settle the issue is a reason to suspend on *p*” (Schmidt, 2023b, 5). If this is right, then evidentialists can say that, in cases where one lacks sufficient evidence, or where one’s evidence is tied, higher-order evidence about one’s evidential situation provides one with a reason to suspend judgment. Furthermore, they can add that, if one has evidence that one’s cognitive capacities are impaired (for instance, by hypoxia), then this is also evidence that one’s evidence does not settle the issue, which is likewise a reason to suspend.^3^

We might wonder about the weight of these higher-order evidential reasons to suspend and about how they interact and compare with other kinds of epistemic reasons. However, these questions also arise for any view that acknowledges non-evidential reasons for suspension, such as incoherence-based reasons.^4^ So, let us put them aside and return to Basna’s case.

If Schmidt is right in allowing for higher-order evidential reasons to suspend, then Basna *has* evidence that her evidence does not settle the issue: she has testimony from two experts and arguments from two experts that, it seems, roughly balance out. She therefore has a reason to suspend judgment that is provided by evidence that her evidence does not settle the issue. Evidentialists can appeal to this reason to explain why she should suspend judgment.

Since Schmidt acknowledges this reason to suspend, it is unclear what work the incoherence-based reason to suspend can still do. Does it provide an *additional* reason to suspend judgment above and beyond the higher-order evidential reason? This seems to overdetermine suspension as the required response. The incoherence-based reason seems *superfluous* in an explanation of why Basna should suspend judgment. Given that the balance of evidential reasons—which includes higher-order evidence about the evidential situation—already settles that Basna ought to suspend, the incoherence itself does not seem to be normatively relevant.

Schmidt could reply that what *rationally motivates* Basna to suspend is the awareness of her incoherence, rather than her evidence. For on an intuitive reading of the case, Basna becomes aware of her contradictory beliefs, and *in response to this incoherence* suspends judgment about each belief. So, even if there are several kinds of reasons for suspending judgment, the fact about incoherence is Basna’s motivating reason for suspension.

The problem with this reply is that it is epistemically irrational to revise an individual belief merely on the basis of its inconsistency with other beliefs. What is rational is to *disbelieve* a set of inconsistent propositions on the basis of their inconsistency. This is because, on a sufficiently broad conception of evidence, the inconsistency of the set is very strong evidence that the set is false (the likelihood is 1). However, this inconsistency is not evidence against any *particular* belief within the set. It could still be that any particular belief is well-supported by one’s evidence. Yet, it is not possible that their *conjunction* is supported by evidence. This is why we have reason to treat inconsistent beliefs as off-limits in our deliberations (Worsnip, 2021): we can be sure that their conjunction is false.^5^

Insofar as Basna is rational, she cannot be motivated to suspend about a particular belief by an awareness of the incoherence of the set of beliefs to which this belief belongs. Rather, she is motivated by her evidence to disbelieve [*p* and not-*p*]. This evidence is provided by the fact that the conjuncts are inconsistent.

Basna also suspends judgment about *p*. Yet, her suspension is motivated by evidence that her evidence does not settle the issue. It would be *a thought too many* if she were *also* to base her suspension on the incoherence of her beliefs itself. For note that if Basna *were* to notice immediately that the arguments of her philosophy professors are superior to the arguments of her history professors, then she would rationally come to believe not-*p*. This indicates that a fully rational Basna is responsive to her evidence rather than to facts about incoherence in revising her *particular* attitudes.

One might object that Basna does not even remember the evidence for relativism but only *that she believes in relativism*. So, she cannot respond to her higher-order evidence that her evidence does not settle the issue: she does not even know what her evidence was, exactly.

In reply, note that if Basna only remembers her belief in relativism *without* also remembering that her belief had been acquired on the basis of sufficient evidence (whatever it was, exactly), then it would not be rational for her to suspend. Rather, if Basna had serious doubts about whether her belief in relativism had been acquired on the basis of sufficient evidence, then she should now believe that relativism is false (given her philosophy professor’s arguments she now encounters). However, as the case is described, Basna’s earlier acquired belief in relativism had been formed on the basis of sufficient evidence, and presumably, she rationally trusts her former self. So, her first-order evidence for and against *p* is tied. This is what rationally motivates her to suspend.

## Lotteries and epistemic akrasia

I have argued that the incoherence-based reason in Basna’s case is normatively superfluous, because her evidential reasons already favor suspension. It is a delicate question of how to spell out the details of such an evidentialist view of reasons for suspension, especially within a reasons-first framework (I return to this issue briefly in conclusion). For the present purposes, it is enough to point out that Schmidt herself acknowledges that there are higher-order evidential reasons for suspension. If suspension can be rationally motivated by such evidence, then facts about the incoherence of one’s beliefs themselves seem normatively superfluous. I will now consider Schmidt’s lottery and epistemic akrasia cases in light of this argument.

Let’s consider Schmidt’s lottery case first:

*6/49 Lottery*

Lola participates in a lottery in which each player chooses six numbers from 1 to 49, and wins the jackpot if her numbers match the six numbers produced in the drawing. The probability that she will win the jackpot is 1 in 13,983,816. There is no guarantee that anyone will win the jackpot. The lottery has millions of regular participants, and as a matter of fact, the chance in any drawing that at least one player wins the jackpot is extremely high. To fix ideas, say that over the last five decades, there has been only 1 in 1,000 drawings in which no ticket won the jackpot. Lola is aware both of the extremely high chances of losing of every single ticket and, by way of induction, of the extremely low chances that *everyone*’s tickets will lose (not win the jackpot). It is then extremely probable, from Lola’s point of view, for ticket 1, that it will lose; for ticket 2, that it will lose; …; for ticket *n*, that it will lose (call the respective propositions “*p*_*1*_”, “*p*_*2*_”, …, “*p*_*n*_”). But at the same time, it is extremely probable for her that it is *not* the case that ticket 1 will lose *and* that ticket 2 will lose, …, *and* that ticket *n* will lose (call this proposition “*p*_*□*_”). (Schmidt, 2023b, 7–8)

On a probabilistic conception of evidence, Lola’s evidence decisively supports believing each *p*_*1*_, *p*_*2*_, … *p*_*n*_. After all, each individual proposition is extremely likely to be true. Yet, their conjunction is false with a very high probability. So, Lola’s evidence decisively supports believing *p*_*□*_, which is just the negation of the conjunction of all the individual claims. According to Schmidt, Lola should suspend judgment about all propositions, although each is well-supported by her evidence. The reason to suspend is their inconsistency, rather than any evidential reason.

Evidentialists can reply by arguing either that it is not the case that Lola ought to suspend about all propositions, given that they are well-supported by her evidence, or else by arguing that Lola should suspend on the basis of her evidence. Let us consider each direction.

According to the first reply, Lola is permitted to believe *p*_*1*_, *p*_*2*_, … *p*_*n*_* and* permitted to believe *p*_*□*_, although the claims are incompatible. One way to make this intelligible is to say that some contradictions are epistemically harmless: they do not amount to a serious form of epistemic irrationality. Lord (2018, 60) holds this view about the Preface Paradox: suppose you wrote a book, and when considering each of your claims individually, you conclude that your evidence decisively supports each individual claim; however, since you acknowledge your own fallibility, you do not believe the conjunction of all claims that you made in your book. You might even *disbelieve* the conjunction. Yet, you still seem rational. So, this might be a case of *harmless incoherence*: we cannot epistemically criticize or blame you for not revising your belief in response to this kind of incoherence.

One might find this reply implausible in *6/49 Lottery*.^6^ I think evidentialists could alternatively defend the verdict that Lola should suspend judgment. The most straightforward way is to adopt a conception of evidence that is not purely probabilistic.^7^ However, even within a largely probabilistic framework, evidentialists have leeway by appealing to higher-order evidence. For as I have noted in Section 2 above, inconsistency is conclusive evidence that the inconsistent set of beliefs is false. So, Lola’s evidence decisively supports that it is not the case [*p*_*1*_, *p*_*2*_, … *p*_*n*_ and *p*_*□*_]. Yet, her evidence supports each conjunct. This is a confusing situation. So, Lola plausibly has higher-order evidence that her evidence does not settle whether each individual proposition is true. Again, she should suspend—at least if she does not know whether the incoherence is harmless, as in the Preface Paradox.

How does this strategy apply to Schmidt’s final case of epistemic akrasia? Here is her case, modified from Worsnip (2018), in full:

*Marple and Poirot*

Miss Marple and Hercule Poirot team up investigating a murder. Master detective Miss Marple is first on the scene and takes in all the evidence, forming the (for once, mistaken) belief that the evidence indicates *that the vicar did it* (*v*), and she tells Poirot so. That Miss Marple provides this testimony is a sufficient reason for Poirot to believe that the evidence indicates that *v*, and he forms the belief for that reason. Next, Poirot himself takes in the evidence at the crime scene, which as a matter of fact indicates that the vicar didn’t do it; he therefore has sufficient reason to disbelieve *v*, and disbelieves *v* for that reason. Poirot now has incoherent doxastic attitudes, belief that the evidence indicates that *v* and disbelief that *v*. They are incoherent because by virtue of his belief about the evidence, he accepts that there is sufficient evidence and thus reason to believe that *v*, and thus that belief that *v* is the correct doxastic response; but nonetheless, he disbelieves *v*. (Schmidt, 2023b, 10)

According to Schmidt, when Poirot finds himself in this epistemic situation, the incoherence of his attitudes “is a *pro tanto* epistemic reason for him to take a step back from the whole mess and to reassess his epistemic situation—i.e., a reason to suspend on both propositions,” which is a reason that “outweighs the evidential reasons backing these attitudes” (Schmidt, 2023b, 10).

Interestingly, Schmidt adds that Poirot’s reason to suspend is sufficient “at least as long as he has no full view of his evidence-based reasons and their quality” (Schmidt, 2023b, 10). But this suggests that he has higher-order evidence that his evidence, *as far as he knows it*, is insufficient. Schmidt here commits to the view that one’s evidence could be *elusive*: one might not be in a position to know what it supports, at least not without further inquiry. So, if one’s evidence cannot settle the question at the moment, then incoherence can at least settle one’s doxastic attitudes.

One reply is to reject that elusive evidence is relevant for what doxastic attitude one ought to adopt. Rather, it is one’s evidence *insofar as one knows it* that is relevant. Evidentialists can plausibly appeal to higher-order evidence that Poirot’s evidence, insofar as he knows it, does not settle the matter to explain why Poirot should suspend judgment.

Additionally, it is worth noting that Poirot has been irrational in disbelieving *v* on the basis of his first-order evidence, given that he has *previously* acquired sufficient higher-order evidence that supports *v*. Rather, when he uncovers first-order evidence that not-*v*, he should immediately come to suspend based on the overall balance of evidential reasons.

## Conclusion

For every case of incoherent beliefs that Schmidt presents, we can either show that there was higher-order evidence that should rationally motivate the person to suspend about an *individual* proposition (for incoherence is only evidence against incoherent *sets* of propositions), or, in some cases, the incoherence might be epistemically harmless. It would be irrational to be motivated by harmless incoherence to suspend judgment, at least if one knows that it is harmless. The latter might be plausible in the Preface Paradox, and so maybe also in *6/49 Lottery*.

Note that my argument mirrors a common strategy employed by theorists of rationality who argue that rationality is not a matter of coherence but rather a matter of responding correctly to reasons (Kiesewetter, 2017, chs. 9–10; Lord, 2018, 26–61). Roughly, the strategy goes as follows: for any *irrationally* incoherent set of attitudes, it is true that one’s reasons fail to support at least one of these attitudes, and so irrational incoherence *guarantees* that one fails to respond correctly to one’s reasons. There will be some exceptional cases in which incoherent attitudes do not involve a failure to respond to reasons—but it can then be argued that these are not cases of *irrationality*. On my favored reading, these are cases of *harmless incoherence* (Schmidt, forthcoming, ch. 3). This strategy is important as a first step for reducing the irrationality of incoherence to failures of responding to reasons.^8^ As we saw, similar moves can help evidentialists to deny that incoherence provides epistemic reasons for suspension.

Importantly, I agree with Schmidt (2023b, 15–18) that the incoherence itself does not *reduce* to higher-order evidence. Rather, my point is that the incoherence itself is not a reason *in addition* to the higher-order evidence in each of Schmidt’s cases. For it is irrational to revise any particular belief merely on the basis of incoherence. Rational agents are instead responsive to their overall evidential situation. Suspending judgment on the basis of higher-order evidence is what allows the subjects to reopen deliberation.^9^

Schmidt begins her article by noticing that her project renders reasons-first epistemology interesting: if epistemic reasons are not just provided by evidence, then talk about reasons and talk about evidence will come apart. This is why thinking about epistemological issues in terms of reasons can give us interesting new insights.

By contrast, I think that reasons-first epistemology becomes interesting because it forces us to think more precisely about *how* evidence provides reasons for belief. But I do not think that this should ultimately lead us to reject evidentialism. I would like to conclude this commentary with a remark about reasons-first *evidentialist* epistemology.

Note that as soon as evidentialists acknowledge that the *balance of reasons* determines what doxastic attitude one should epistemically have, evidence-talk will not straightforwardly translate into reasons-talk. In cases where the evidence for and against *p* is equally balanced, one’s epistemic reasons for and against believing *p* are not equally weighty. Rather, one’s reasons against belief are weightier than one’s reasons for belief, and one’s reasons for suspension are weightier than one’s reasons for belief and weightier than one’s reasons for disbelief. So, even if all epistemic reasons are provided by evidence, evidence and epistemic reasons still intuitively come apart (see Schroeder, 2021).

On a reasons-first evidentialist view, the overall balance of *evidential* reasons explains why one ought to suspend judgment in cases of tied evidence. It is surely a challenge for evidentialism to make sense of this verdict. However, evidentialists could meet this challenge by showing how higher-order evidence and first-order evidence combine to determine the overall balance of epistemic reasons in various cases, including cases of tied evidence. This project was not clearly visible until the reasons-first movement arose.

Importantly, the kind of higher-order evidence that was central to my argument here—evidence that the evidence is insufficient—cannot be the *ultimate* source of epistemic reasons against belief in a reasons-first epistemology, for it is evidence that the evidence is insufficient to *justify* or *rationalize* belief. But reasons-first proponents cannot appeal to other normative notions than the notion of a reason in analyzing sufficiency—this would mean giving up on the reasons-first project. So, right now, we lack an account of how evidential reasons alone could determine the overall balance of epistemic reasons. However, this does not mean that such an account cannot be developed.^10^

## Footnotes

1 This is because “the cause of the incoherence is quite harmless” (Schmidt, 2023, 12). The incoherence is caused by compartmentalization.

2 Berker (2018) argues that epistemic reasons admit of *prohibitive balancing*: when the epistemic reasons for and against believing *p* are equally weighty, then one is prohibited from believing *p* and prohibited from disbelieving *p*. Schroeder (2021) and Brunero (2022), by contrast, commit to *permissive balancing*: equally weighty reasons for and against an option make the option permissible. Permissive balancing is more promising for reasons-first views, which must hold that the *overall balance of reasons* determines what doxastic attitude is *rational* or *justified*. Permissive balancing seems to be a promising analysis of this commitment of reasons-first views. On Berker’s prohibitive balancing view, by contrast, suspension seems to be required only because (dis)belief is prohibited, not because the reasons against belief or for suspension are weightier (which would just bring us back to permissive balancing).

3 Schmidt (2023, 6) says that such higher-order evidence functions as an undercutting defeater of the first-order evidence. I think this is plausible in cases of evidence for *severe* cognitive impairment, but not when one has evidence that one’s capacities are only *slightly* impaired. In the latter cases, the first-order evidence does not lose all its normative force. The higher-order evidence is here plausibly a reason to suspend. Schmidt could draw on her view about pragmatic encroachment (Schmidt, 2023a) and argue that such higher-order evidence *attenuates* the weight of the first-order evidential reasons, rather than providing a reason to suspend. However, this does not capture cases where, say, one’s mathematical reasoning seems completely sound even after rechecking many times, but one learns that one suffers a slight impairment in one’s mathematical abilities. Here, one’s first-order evidence seems as strong as it can be, but one gets a reason to suspend when one learns about the impairment.

4 One might even argue that the issue is more pressing for these views: plausibly, there is some kind of weighing interaction between higher-order evidence and first-order evidence; but it is unclear how we should weigh facts about incoherence or other state-given reasons with first-order evidence.

5 For this reason, Basna’s beliefs are no longer supported by *sufficient* evidence when she notices the inconsistency (the evidence on both sides is merely “good” or “strong” but *insufficient*): clearly, both beliefs cannot be true.

6 Schmidt (2023, 18; fn. 20) notes that there might be disanalogies between the two cases. You might think that you are clearly prohibited from believing each lottery proposition, even though it is highly likely to be true. This can give rise to an objection against a probabilistic version of evidentialism. But it is not Schmidt’s argument.

7 Schmidt (2023, 6–7) points out that a probabilistic conception of evidence allows evidentialists to say that the fact that is believed can be a reason to believe *p* in cases of perceptual belief. See also Schmidt (2021) and Williamson (2000, 186–187).

8 Importantly, it is not the whole way to such a reduction. For it might still be that, even though one always fails to respond correctly to reasons when one’s attitudes are irrationally incoherent, there can still be an additional and *sui generis* mistake of attitudinal incoherence in such cases (Worsnip, 2021, 5). Furthermore, a mere *implication* from incoherence to failures of reasons-responsiveness does not show that irrational incoherence *reduces* to reasons-responsiveness. I stay neutral about whether coherence is normative in the way Worsnip argues: as providing right-kind reasons for deliberating in such a way that one treats incoherent sets of attitudes as off-limits.

9 As an anonymous reviewer has helpfully pointed out, the requirement to revise one’s beliefs on the basis of the incoherence itself could at best be a *wide-scope* requirement: if one believes *p* and not-*p*, then incoherence would require one to [not believe *p or* not believe not-*p*]. This is another way to put my point that incoherence provides a decisive evidential reason against believing [*p* and not-*p*], but not a reason against any individual belief.

10 In a current project, I take some steps towards developing such a view. For an account that is compatible with reasons-first and more friendly to evidentialism than Schroeder’s view, see Brunero (2022).
